# Osilodrostat Safety Profile: Findings from Real-World Data in the FAERS Database

**DOI:** 10.3390/jcm14103518

**Published:** 2025-05-17

**Authors:** Ioana Rada Popa Ilie, Anca Butuca, Calin Homorodean, Carmen Maximiliana Dobrea, Claudiu Morgovan, Adina Frum, Steliana Ghibu

**Affiliations:** 1 Department of Endocrinology, Faculty of Medicine, “Iuliu Haţieganu” University of Medicine and Pharmacy, 3-5 Louis Pasteur Street, 400349 Cluj-Napoca, Romania; ioana.ilie@umfcluj.ro; 2 Preclinical Department, Faculty of Medicine, “Lucian Blaga” University of Sibiu, 550169 Sibiu, Romania; carmen.dobrea@ulbsibiu.ro (C.M.D.); claudiu.morgovan@ulbsibiu.ro (C.M.); adina.frum@ulbsibiu.ro (A.F.); 3 Medical Clinic No. 1, Internal Medicine Department, University of Medicine and Pharmacy “Iuliu Hatieganu”, 400006 Cluj-Napoca, Romania; 4 Interventional Cardiology Department, Cluj County Emergency Hospital, 400006 Cluj-Napoca, Romania; 5 Department of Pharmacology, Physiology and Pathophysiology, Faculty of Pharmacy, “Iuliu Haţieganu” University of Medicine and Pharmacy, 6A Louis Pasteur Street, 400349 Cluj-Napoca, Romania; steliana.ghibu@umfcluj.ro

**Keywords:** osilodrostat, adverse effect, pharmacovigilance, disproportionality analysis, FAERS database

## Abstract

**Background/Objectives:** Cushing’s syndrome (CS), including Cushing’s disease (CD)—the most common type—has a substantial negative impact on morbidity, mortality, and patients’ quality of life. Medical management of CS is essential for controlling hypercortisolism as part of preoperative preparation for definitive surgical treatment and for managing residual or relapsed hypercortisolism post-surgery. Osilodrostat, a dual inhibitor of glucocorticoid and mineralocorticoid biosynthetic pathways, has been approved for the medical treatment of CS since early 2020. However, real-world data on its adverse effects remain limited. We mined the FAERS database and analyzed the reports associated with osilodrostat up to 1 October 2024. **Methods:** Descriptive and disproportionality methods based on Relative Odds Ratio (ROR), Chi-square (χ^2^), and Proportional Reporting Ratio (PRR), were used to discern potential safety signals and assess the significance of osilodrostat-associated adverse events. **Results:** This study identified 782 reports in which osilodrostat was the primary suspected drug, containing 593 preferred terms (PTs) and 2481 occurrences. The most frequently registered events belonged to the following SOCs: “General disorders and administration site conditions” (*n* = 457, 18.4%), “Injury, poisoning and procedural complications” (*n* = 311, 12.5%), “Gastrointestinal disorders” (*n* = 278, 11.2%), “Investigations” (*n* = 260, 10.5%), and “Nervous system disorders” (*n* = 184, 7.4%). Among PTs, off-label use was the most commonly reported, aligning with the fact that the vast majority of cases originated from the U.S. (84%), where osilodrostat is officially approved only for the treatment of CD. Disproportionality analysis confirmed previously known and new potential adverse drug reactions associated with osilodrostat treatment, including reports of cardiac flutter (*n*: 4; PRR: 19.42; χ^2^: 49.57), ventricular extrasystoles (*n*: 4; PRR: 11.85; χ^2^: 29.62), muscular weakness (*n*: 8; PRR: 2.25; χ^2^: 4.38), rib fracture (*n*: 4; PRR: 6.66; χ^2^: 13.99), spinal fracture (*n*: 3; PRR: 4.66; χ^2^: 5.35), sepsis (*n*: 9; PRR: 2.63; χ^2^: 7.56), fungal infections (*n*: 4; PRR: 3.67; χ^2^: 5.33), and COVID-19 (*n*: 32; PRR: 5.07; χ^2^: 101.16). **Conclusions:** This study highlights new risks and offers valuable insights into osilodrostat use; however, further research and validation are necessary, particularly for adverse reactions not yet explicitly documented in the summary of product characteristics.

## 1. Introduction

Cushing’s syndrome (CS) is a rare but serious endocrine disorder characterized by chronic hypercortisolism. The most common cause of CS is pituitary corticotropinoma [Cushing’s disease (CD)], an ACTH-dependent form of CS, in which the pituitary tumor secretes ACTH autonomously, driving excessive cortisol production by the adrenal glands. CD accounts for approximately 80% of endogenous CS cases. Less frequently, CS can result from ectopic ACTH secretion from non-pituitary tumors or from ACTH-independent cortisol production, typically caused by adrenal lesions such as adenomas, nodular hyperplasia, or carcinomas [1,2]. Osilodrostat, a potent oral inhibitor of 11β-hydroxylase, an enzyme involved in cortisol synthesis, was approved by the European Medicines Agency (EMA) on 9 January 2020, and by the United States (US) Food and Drug Administration (FDA) on 6 March 2020, for the treatment of patients with CS (EMA) and CD (FDA), respectively. It is indicated for individuals who are either ineligible for pituitary surgery or have experienced surgical failure [3,4].

Surgery, aiming to remove the primary tumor, remains the mainstay of therapy in CS regardless of etiology and should be pursued whenever feasible—even in cases of malignancy, although it is rarely curative in such scenarios.

In CD, remission rates following transsphenoidal surgery range from 65% to 90%, but hypercortisolism recurs in approximately 2% of patients annually, with a long-term relapse rate of up to 30% [5,6,7,8,9,10]. Consequently, many patients require additional treatment, which may include repeat surgery, pituitary radiation therapy, medical therapy, or bilateral adrenalectomy. The primary goal of all interventions remains biochemical control and normalization of cortisol levels [9,11]. Currently, available medications for CD fall into three categories: pituitary-targeted therapies (e.g., pasireotide), adrenal-directed medications (e.g., metyrapone), and a glucocorticoid receptor antagonist (e.g., mifepristone) [1].

Adrenally directed medications are steroidogenesis inhibitors that block one or more steps in cortisol synthesis. The currently available options include ketoconazole, levoketoconazole, metyrapone, osilodrostat, mitotane, and etomidate. It is important to note that in the U.S., the use of ketoconazole, metyrapone, mitotane, or etomidate for patients with CD is considered off-label [12]. On the other hand, severe hypercortisolism (SH) resulting from ectopic ACTH syndrome (EAS) or adrenocortical carcinoma (ACC), which requires rapid cortisol normalization, may also benefit from steroidogenesis inhibitors such as ketoconazole and metyrapone, either as monotherapy or in combination therapy.

Osilodrostat inhibits 11β-hydroxylase [cytochrome P450 (CYP) 11B1] and also blocks aldosterone synthase (CYP11B2, steroid 18-hydroxylase). These enzymes play a key role in the final steps of cortisol synthesis and aldosterone synthesis in the adrenal gland. By inhibiting CYP11B1, osilodrostat effectively reduces and normalizes cortisol levels. However, this inhibition triggers a compensatory increase in ACTH secretion through feedback mechanisms, leading to the accumulation of precursors such as 11-deoxycortisol and enhanced adrenal biosynthesis, including increased androgen production [13].

In the US, the incidence of CD is estimated to be 6.2 to 7.6 cases per million patient-years across all populations. However, among women aged 18 to 24 years, the incidence is higher, ranging from 16.7 to 27.2 cases per million patient-years [14]. Patients with uncontrolled CD face a significant economic burden, with healthcare-related costs averaging around USD 30,000 per year and lifetime expenses exceeding USD 880,000 due to comorbidities associated with the condition [15]. This underscores the need for effective drugs to control active CD. Although clinical trials have confirmed the efficacy of osilodrostat in CD, its recent introduction to the market raises concerns about adverse drug reactions (ADRs). Therefore, post-marketing surveillance and real-world data are essential for a more comprehensive understanding of its safety profile in routine clinical practice. Ongoing monitoring and evaluation of new medications are particularly important to refine treatment recommendations and ensure patient safety.

FAERS (FDA Adverse Event Reporting System), one of the largest pharmacovigilance databases in the world, contains reports on drug adverse events and medication errors submitted to the FDA [16]. It serves as a valuable resource for assessing ADRs in a real-world setting [17]. By analyzing FAERS data, we aim to provide a comprehensive evaluation of the safety profile of osilodrostat, identifying the most frequently reported adverse effects, their severity, and any emerging safety signals that may differ from clinical trial findings.

## 2. Materials and Methods

### 2.1. Study Design and Data Extraction

In the FAERS database, safety spontaneous reports from different countries by various categories of reporters (healthcare professionals, patients, pharmaceutical manufacturers, etc.) are collected. In order to report an adverse event in FAERS several terms could be used. According to MedDRA dictionary (version 27.1) their classification could be performed by using different levels: low level term (LLT), preferred term (PT), high level term (HLT), high level group term (HLGT), and System Organ Class (SOC). PT is the standard term used to report a specific ADR. It is used to identify pharmacovigilance signals which represent ADRs unlisted in the summary of product characteristics. Information from FAERS can be accessed through OpenVigil 2.1, a tool that contains curated data derived from valid reports [18,19].

The safety profile of osilodrostat was assessed by analyzing the proportional imbalance of adverse events. Data mining was conducted using the OpenVigil 2.1 tool. Reports submitted to the FAERS database up to 1 October 2024, were analyzed. Only reports in which osilodrostat was classified as the “Primary Suspect Drug” were considered, while those where osilodrostat was listed as a “Secondary Suspect Drug”, “Concomitant”, or “Interacting” were excluded. All reports that included “osilodrostat” as the drug name were exported from OpenVigil 2.1. Duplicate reports with the same “Case_id” were removed using the “Remove Duplicates” function in Microsoft Excel for Microsoft 365 MSO (Version 2503). The selection of the Role Code was based on the “Primary Suspect Drug” designation.

### 2.2. Descriptive Analysis

The curated data, obtained after the extraction and processing steps, were used for descriptive analysis. The distribution of cases was analyzed based on role (“Primary Suspect Drug”, “Secondary Suspect Drug”, “Concomitant”, and “Interacting”), year, region, country, gender (male, female, not specified), and unfavorable outcomes (“Life-threatening”, “Hospitalization—Initial or Prolonged”, “Disability”, or “Death”). Subsequently, the 30 most frequently reported PTs were identified.

### 2.3. Disproportionality Analysis

Disproportionality analysis was used to identify potential safety signals, where a suspected ADR was disproportionately represented in a database for a specific drug. The signal detection method is based on a two-by-two contingency table (Supplementary Materials—Table S1), which compares the frequency of the suspected ADR with other events in the database [18]. However, further pharmacological studies are required to confirm the results obtained through the disproportionality method.

First, the reporting rate (R), a simple, but insufficient parameter for signal detection, was calculated. A positive signal in FAERS could be considered if the following criteria were met:

(i) Number of cases > 2;
(ii) Chi-square (χ^2^) > 4;
(iii) Proportional Reporting Ratio (PRR) > 2 [20].

In addition to Evans’ criteria, we used the reporting odds ratio (ROR) and the 95% confidence interval (95% CI) to evaluate disproportionate signals (Supplementary Materials—Table S2).

### 2.4. Ethics

The data used in this study did not contain patient-identifiable information; therefore, no ethical approval was required.

## 3. Results

### 3.1. Data Mining

**Figure 1:**
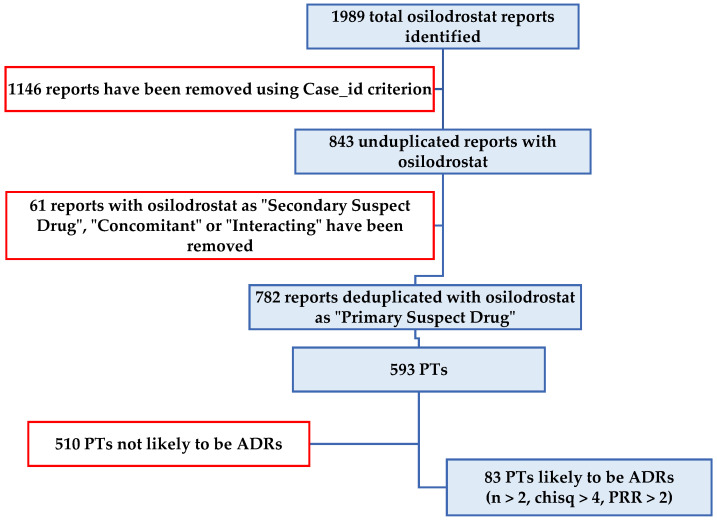
Flow chart of data selection for osilodrostat in the FAERS database up to 1 October 2024. ADR—adverse drug reaction; PRR—proportional reporting ratio; PT—preferred term. Data excluded are indicated in red frames.

The flow chart of data selection for osilodrostat in the FAERS database up to 1 October 2024 is shown in Figure 1.

**Figure 2:**
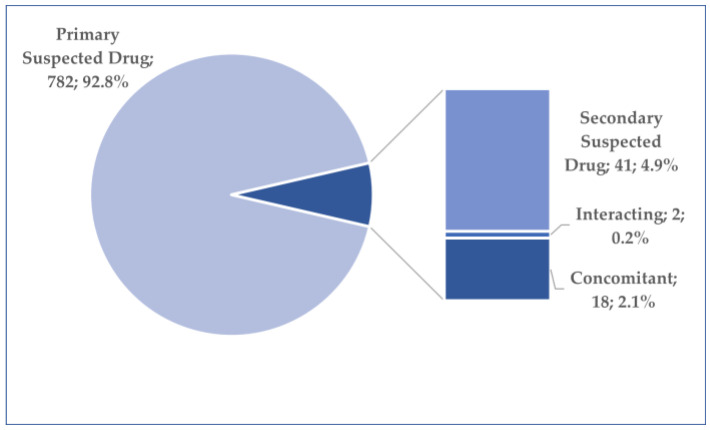
Distribution of cases by role in ADR occurrence.

Out of a total of 1989 reports related to osilodrostat use, 843 reports were deduplicated (using the “Case_id” criterion). A total of 61 reports related to roles other than “Primary Suspect Drug” were excluded (Figure 2).

For the remaining 782 reports where osilodrostat was identified as the “Primary Suspect Drug”, 593 PTs were detected. After conducting the disproportionality analysis based on the criteria established by Evans, only 83 PTs met the criteria for being considered probable ADRs.

### 3.2. General Characteristics of Cases Related to Osilodrostat Use

**Figure 3:**
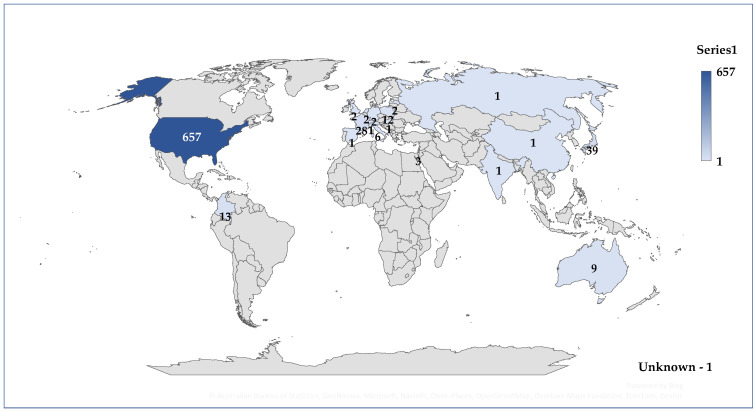
Distribution of cases by country of origin. UNK—unknown country.

**Figure 4:**
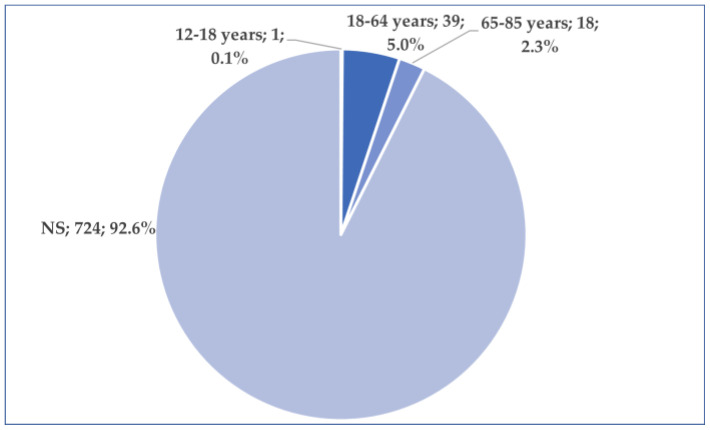
Distribution of cases by age. NS—not specified.

**Figure 5:**
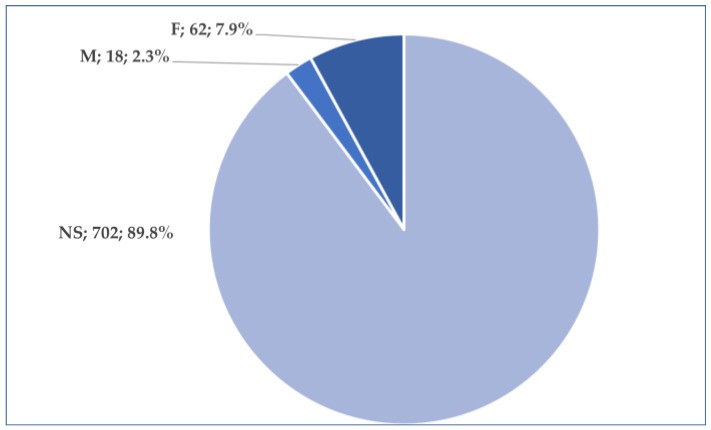
Distribution of cases by gender. F—female; M—male; NS—not specified.

**Figure 6:**
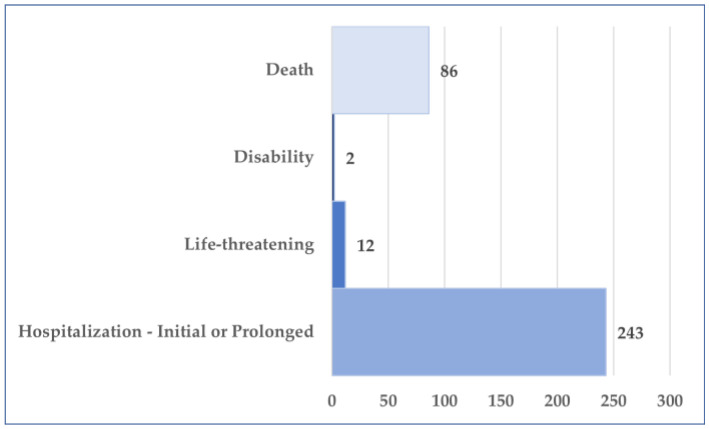
Distribution of cases with an unfavorable outcome.

More than 84% of the total reports (*n* = 657, 84.0%) originated from the US. A total of 58 cases (7.4%) were reported from Europe, while 44 cases (5.6%) came from Asia. After the US, the second-highest number of reports came from Japan (*n* = 39), followed by France (*n* = 28) (Figure 3). According to the results presented in Figure 4, age is mentioned in only 7.4% of the total cases. The average patient age is 52.4 years. The majority of these patients belonged to the 18–64 years age group (*n* = 39, 5.0%), while a smaller proportion (*n* = 18, 2.3%) fell within the 65–85 years age group. Regarding gender distribution, approximately 90% of the reports did not include the patient’s gender. In the remaining 10% of cases where gender was specified, the female-to-male ratio was 3.4:1 (Figure 5). In 318 cases, at least one unfavorable outcome was reported. Figure 6 illustrates the distribution of cases by outcome.

The most frequently reported unfavorable outcome was hospitalization—initial or prolonged (*n* = 243). Hospitalization was required in 31.1% of total cases, regardless of whether it was an initial or prolonged admission. Additionally, in 86 reports (accounting for 11.0% of total cases) where osilodrostat was identified as the primary suspect drug for adverse reactions, patient death was recorded.

### 3.3. Analysis of Prefered Terms Distribution

Osilodrostat was the primary suspected drug in 2481 occurrences. The most frequently registered events belonged to the following SOCs: “General disorders and administration site conditions” (*n* = 457, 18.4%), “Injury, poisoning and procedural complications” (*n* = 311, 12.5%), “Gastrointestinal disorders” (*n* = 278, 11.2%), “Investigations” (*n* = 260, 10.5%), and “Nervous system disorders” (*n* = 184, 7.4%) (Supplementary Materials—Table S3).

**Figure 7:**
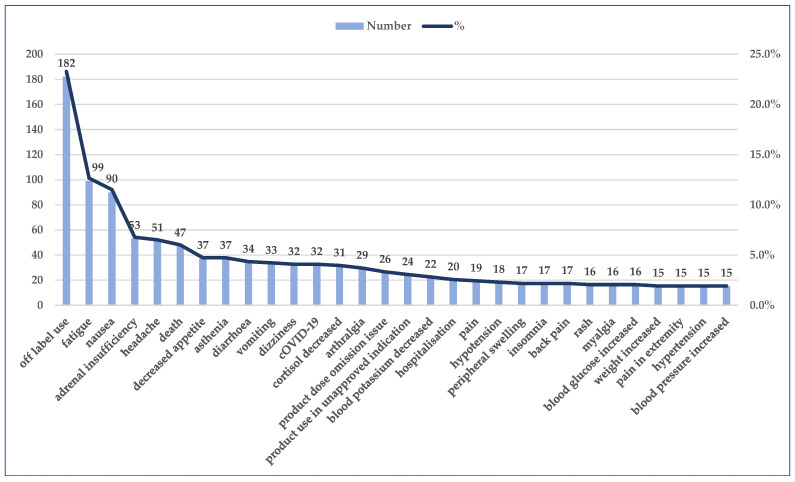
The most frequent 30 PTs and the reporting rate. PT—preferred term, %—reporting rate (R).

Out of a total of 782 cases, the highest reporting rates (R) were registered for the following PTs: off label uses (*n* = 182, R: 23.3%), fatigue (*n* = 99, R: 12.7%), nausea (*n* = 90, R: 11.5%), adrenal insufficiency (*n* = 53, R: 6.8%), and headache (*n* = 51, R: 6.5%) (Figure 7).

### 3.4. Disproportionality Analysis

According to the criteria established by Evans [20], 83 PTs could be considered likely ADRs (Supplementary Materials Table S4). The Proportional Reporting Ratio (PRR) is considered an indicator of the frequency of reporting a PT for a drug of interest. The 10 PTs with the highest PRR have been included in the following SOCs:

(i) Endocrine disorders: mineralocorticoid deficiency (PRR: 5513.88), hyperadrenocorticism (PRR: 621.28), glucocorticoid deficiency (PRR: 248.13); adrenocortical insufficiency acute (PRR: 154.92)
(ii) Investigations: cortisol free urine increased (PRR: 2005.05), blood corticotrophin increased (PRR: 1140.80), cortisol decreased (PRR: 594.20), cortisol increased (PRR: 497.49), cortisol abnormal (PRR: 529.33)
(iii) Neoplasms benign, malignant and unspecified (incl cysts and polyps): adrenal gland cancer (PRR: 274.78).

On the other hand, the highest Chi-squared (*χ*^2^) values, which indicate the greatest differences in reporting, were obtained for the following PTs: cortisol decreased (*χ*^2^: 17154.684), blood corticotrophin increased (*χ*^2^: 9613.789), mineralocorticoid deficiency (*χ*^2^: 8611.485), adrenal insufficiency (AI) (*χ*^2^: 7523.983), cortisol free urine increased (*χ*^2^: 5470.302), cortisol increased (*χ*^2^: 5300.539), hyperadrenocorticism (*χ*^2^: 4195.851), cortisol abnormal (*χ*^2^: 1564.027), adrenocortical insufficiency acute (*χ*^2^: 1215.394), and adrenal gland cancer (*χ*^2^: 1085.843).

**Figure 8:**
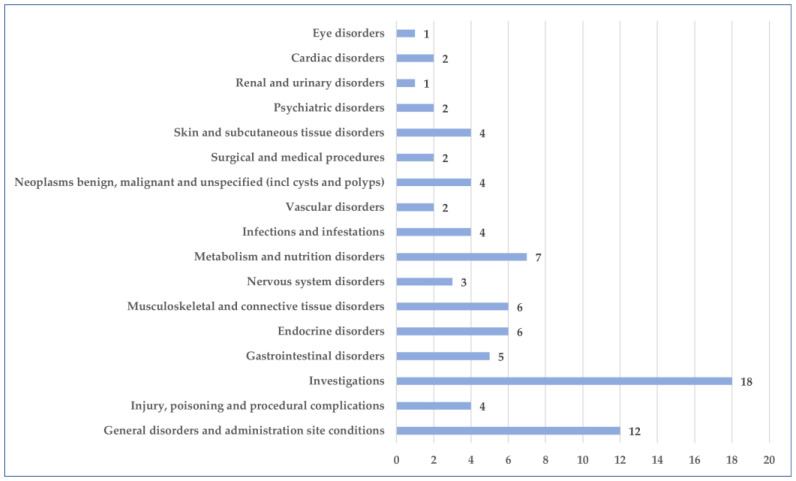
Distribution of PTs by SOC.

Probable ADRs have been reported in 17 SOCs. The most common SOCs were “General disorders and administration site conditions” (12 PTs), “Injury, poisoning and procedural complications” (4 PTs), “Investigations” (18 PTs), “Gastrointestinal disorders” (5 PTs), “Endocrine disorders” (6 PTs), “Metabolism and nutrition disorders” (7 PTs) (Figure 8).

**Figure 9:**
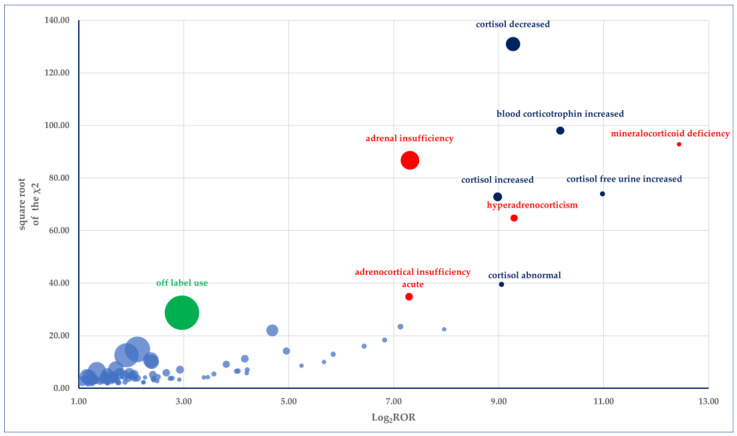
Graphical representation of the strength of probable ADRs related to osilodrostat. The square root of the χ^2^ value is plotted on the *Y*-axis, and Log_2_ROR is plotted on the *X*-axis. The size of each bubble corresponds to the number of reports for each PT. Each bubble represents a probable ADR, with higher and more distant bubbles indicating stronger relations for osilodrostat use. The red color represents PTs from ‘Endocrine Disorders’, green represents PTs from ‘Injury, Poisoning, and Procedural Complications’ SOC, dark blue represents PTs from ‘Investigations’ SOC, and light blue represents all other PTs.

The most significant probable ADRs reported for osilodrostat are found in the “Investigations” SOC (including decreased cortisol, increased blood corticotrophin, increased free cortisol in urine, elevated cortisol, and abnormal cortisol levels), the “Endocrine Disorders” SOC (encompassing mineralocorticoid deficiency, adrenal insufficiency, hyperadrenocorticism, and acute adrenocortical insufficiency), and the “Injury, poisoning and procedural complications” SOC (off-label use) (Figure 9).

**Figure 10:**
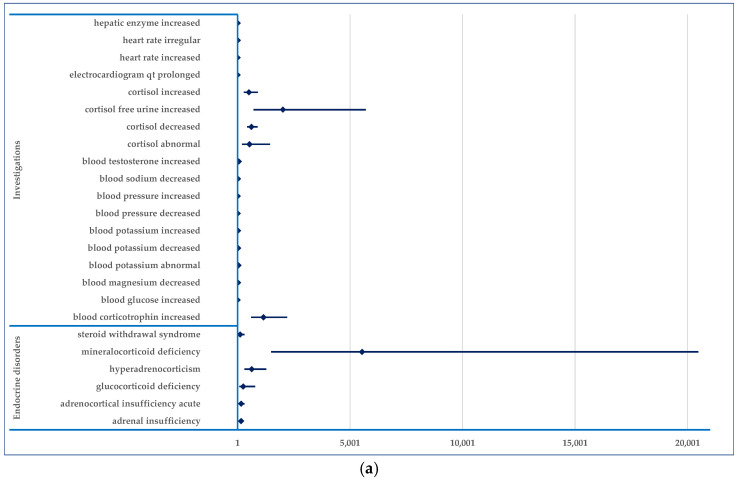

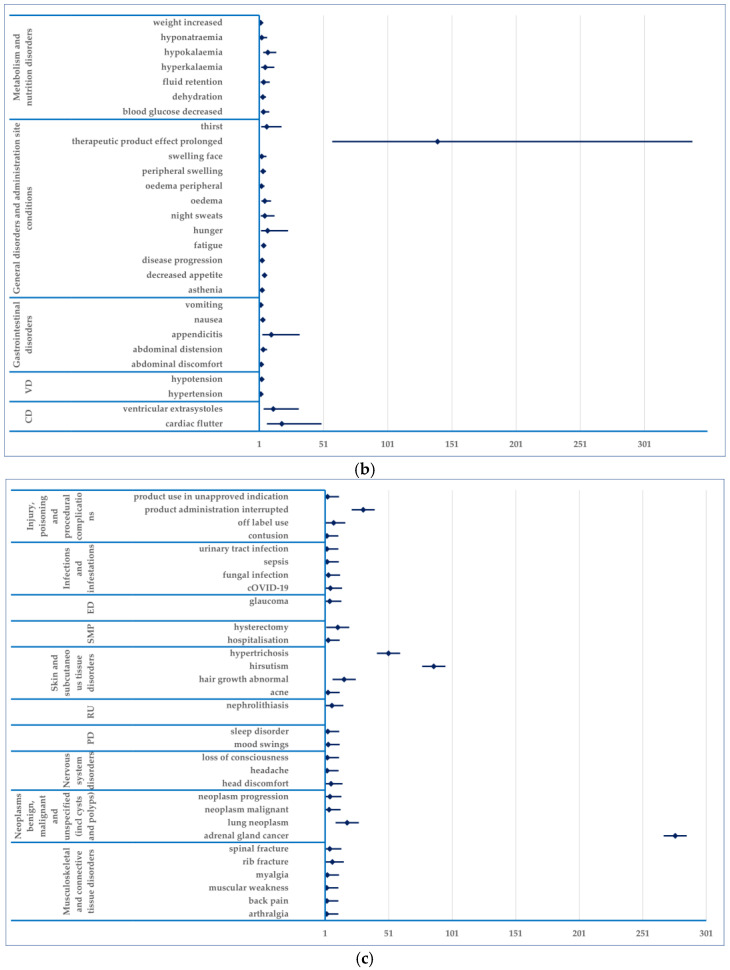
Reporting odds ratio for probable ADRs. (**a**)—“Investigations” and “Endocrine disorders”; (**b**)—“Cardiac disorders”, “Vascular disorders”, “Gastrointestinal disorders”, “General disorders and administration site conditions” and “Metabolism and nutrition disorders”; (**c**)—“Musculoskeletal and connective tissue disorders”, “Neoplasms benign, malignant and unspecified (incl cysts and polyps)”, “Nervous system disorders”, “Psychiatric disorders”, “Renal and urinary disorders”, “Skin and subcutaneous tissue disorders”, “Surgical and medical procedures”, “Eye disorders” and “Infections and infestations”, “Injury, poisoning and procedural complications”. CD—Cardiac disorders; ED—Eye disorders; PD—Psychiatric disorders; RD—Renal and urinary disorders; SMP—Surgical and medical procedures; VD—Vascular disorders.

In the “Investigations” SOC, the modification of certain parameters represents probable ADRs: hepatic enzyme increased, cortisol increased, cortisol decreased, cortisol abnormal, cortisol free urine increased, blood pressure increased, blood pressure decreased, blood potassium increased, blood potassium decreased, electrocardiogram QT prolonged, etc. Additionally, several endocrine disorders could be considered ADRs to osilodrostat, such as mineralcorticoid deficiency, hyperadrenocorticism, adrenal insufficiency, adrenocortical insufficiency acute, glucocorticoid deficiency, and steroid withdrawal syndrome (Figure 10a).

In the “Cardiac disorders” SOC, cardiac flutter and ventricular extrasystoles have been reported for osilodrostat with a higher probability than for all other drugs. Hypertension and hypotension, as vascular disorders, were also reported. Among musculoskeletal and connective tissue disorders, patients treated with osilodrostat had a higher probability of experiencing arthralgia, back pain, myalgia, muscular weakness, rib fractures, and spinal fractures (Figure 10c) compared to other drugs. Likewise, gastrointestinal issues such as abdominal discomfort, distension, nausea, and vomiting were reported with a higher probability for osilodrostat than for other treatments. Additionally, general disorders—including asthenia, decreased appetite, fatigue, edoema, peripheral edoema, and thirst—were more commonly reported. In the “Metabolism and nutrition disorders” SOC, a higher reporting probability was observed for blood glucose decrease, dehydration, fluid retention, hyperkalemia, hypokalemia, and other metabolic imbalances (Figure 10b). According to Figure 10c, some neoplasms have been reported with a higher probability for osilodrostat, such as adrenal gland cancer and lung neoplasm. Certain nervous system disorders (headache, loss of consciousness) and psychiatric disorders (mood swings, sleep disorders) could be considered ADRs. Additional probable ADRs include acne, hirsutism, hypertrichosis, glaucoma, fungal infections, urinary tract infections, sepsis, and COVID-19. Lastly, osilodrostat was used off-label with a higher probability than all other drugs (Figure 10c).

## 4. Discussions

The management of CD and other forms of CS poses significant diagnostic and therapeutic challenges [15,21,22]. Pharmacological therapy plays a key role, particularly as an adjuvant when surgery is incomplete, in recurrent cases, for patients with surgical contraindications, or as preoperative preparation [22,23]. Given the need for improved medical treatments in CD, osilodrostat—a potent 11-β-hydroxylase inhibitor—has emerged as a promising option. Clinical trials have demonstrated its rapid onset of action, long-term cortisol control, sustained biochemical efficacy, and benefits in alleviating clinical signs, physical manifestations of hypercortisolism, and patient-reported outcomes [24].

According to the drug leaflet and clinical trials, the most common adverse reactions (incidence > 20%) include AI, fatigue, nausea, headache, and edema. Some adverse reactions observed with a frequency of more than 10% in a 48-week clinical study in patients with CD were nasopharyngitis, vomiting, arthralgia, back pain, dizziness, abdominal pain, myalgia, hypokalemia, abnormal hormone levels, hypotension, urinary tract infection, increased blood testosterone, and pyrexia. Other notable adverse reactions that occurred with a frequency of less than 10% included hirsutism (9.5%), acne (8.8%) and hypertrichosis in female patients, dyspepsia (8%), insomnia (8%), anxiety (7.3%), depression (7.3%), gastroenteritis (7.3%), malaise (6.6%), tachycardia (6.6%), alopecia (5.8%), increased transaminases (4.4%), a dose-dependent effect on electrocardiogram QT prolongation (3.6%), and syncope (1.5%) [25,26,27]. These ADRs primarily stem from the drug’s mechanism of action.

In our study, though gender and age information were largely missing from the reports, among those that included gender details (10%), the female-to-male ratio was 3.4:1, aligning with findings in the literature. It is well-established that CD predominantly affects females, with reported female-to-male ratios ranging from 3:1 to 5:1 [28].

The primary severe outcomes include hospitalization, life-threatening incidents, disability, or death. At least one unfavorable outcome was reported in approximately 40% of cases, with hospitalization (either initial or prolonged) being the most frequently reported outcome. Death was reported in 86 cases (11% of the total), but a disproportionate signal was not detected, thus it is unlikely to be considered an ADR. This rate is higher than that observed in clinical trials [26,27,29,30,31]. However, considering that CD itself—including patients in remission—carries a high mortality rate [32], this finding may not be unexpected. The estimated mortality rate in CD patients ranges from 9% (observed in a cohort of 346 patients with either persistent disease or in remission) [33] to as high as 21% in a cohort of 419 patients in remission, while it reaches up to 55% in the group of 40 patients who were not in remission, as revealed by a Swedish Nationwide study [34].

The most commonly reported PTs were off-label use, fatigue, nausea, AI, and headache. Disproportionality analysis further indicated that osilodrostat was highly associated with reports coded as off-label use. This aligns with the fact that the majority of cases originate from the U.S., where osilodrostat is officially approved only for the treatment of CD. It is plausible that these ‘off-label use’ reports may reflect the drug’s utilization in other forms of CS, such as EAS, ACC, bilateral nodular disease (BND), benign solitary adenomas, or mild autonomous cortisol secretion (MACS) [35,36,37,38,39,40,41,42,43,44].

AI often presents with hypotension due to volume depletion from reduced mineralocorticoid function, altered mental status, dizziness or fainting, anorexia, vomiting, weight loss, fatigue, recurrent abdominal pain, fever, and hyperkalemia, primarily mediated by hypoaldosteronism. Additional manifestations may include low blood sugar, increased thirst, depression, and irritability. Fatigue, nausea, and headache are therefore common symptoms of AI a well-anticipated side effect that can occur when the dose of steroidogenesis inhibitors exceeds the required amount or if patients experience an intercurrent illness [45,46,47]. The clinical manifestations of glucocorticoid withdrawal syndrome (GWS)—defined as symptoms arising from a reduction in cortisol levels after prolonged pathological elevation—closely resemble those of AI and may include the aforementioned features [35,48]. These are the most frequently reported adverse events of osilodrostat use in CD, as highlighted in the currently available literature [12]. However, all three symptoms were reported less frequently in FAERS than in clinical trials. In the LINC studies 1–4, including the extension phase of LINC 4, the reported incidence rates were as follows: fatigue (28–58%), nausea (32–42%), and headache (25–34.2%) [26,30,31,49,50]. AI requires paraclinical confirmation through serum cortisol measurements, typically showing levels that are low or near the lower limit of normal (LN) (e.g., <5–10 μg/dL [<140–280 nmol/L]; normal range: 5–25 μg/dL [140–690 nmol/L]). The frequency of AI reported in FAERS is also significantly lower than in LINC studies 2, 3, and 4, which assessed the efficacy and safety of osilodrostat in CD, reporting rates between 25% and 32% [12].

Overall, the primary side effects reported in studies on osilodrostat were AI, symptoms and signs associated with AI, or GWS. These occurred with a prevalence as high as 51% during the titration phase but decreased to 6% in the osilodrostat group during the randomized withdrawal phase [51]. In the LINC 3 and LINC 4 trials, hypocortisolism-related adverse eventss were reported in 54.0% and 27.4% of patients with CD, respectively, predominantly during the initial dose titration. These events were generally mild to moderate, with 4% of patients discontinuing treatment for this reason [26,27,30,31]. Notably, these events were investigator-reported, as the study protocol did not mandate a diagnosis of AI based on serum cortisol levels [26,27,31]. The lower incidence observed in LINC 4 may be attributed to the slower dose-escalation schedule (every three weeks vs. every two weeks in LINC 3) [26,30,31]. Additionally, there have been a few reports of prolonged hypocortisolism lasting up to 15 months after osilodrostat discontinuation [52,53], highlighting the need for further research to understand the underlying mechanisms [35].

The disproportionality analysis revealed 83 probable ADRs, representing 14% of the total PTs. Their majority associated with the following SOCs is consistent with the known pharmacological effects of osilodrostat and/or are listed in the currently FDA-approved label: ‘Investigations’, ‘Endocrine disorders’, ‘Vascular disorders’, ‘Gastrointestinal disorders’, ‘General disorders’, and ‘Metabolism and nutrition disorders’. Hence, since osilodrostat can also inhibit aldosterone synthase (CYP11B2), a reduction in blood pressure may be expected in patients with hypertension. However, a compensatory ACTH increase can lead to the accumulation of mineralocorticoid precursors, such as 11-deoxycorticosterone, potentially raising blood pressure and increasing the risk of hypertension, hypokalemia, and edema [9,50,54,55]. As expected, cortisol precursors, including 11-deoxycortisol, accumulate in the blood and urine of patients treated with osilodrostat due to enzymatic blockade. These precursors cross-react with several cortisol immunoassays, leading to falsely elevated or abnormal cortisol levels. To mitigate this issue, cortisol-specific immunoassays or mass spectrometry-based methods should be used [2,12,56]. Osilodrostat, like other 11β-hydroxylase inhibitors (e.g., metyrapone is associated with androgenic side effects (acne and hirsutism) in females [57]), can also increase androgen levels. In the LINC 3 and 4 studies, testosterone initially rose in female patients but gradually returned to baseline with long-term treatment [26,27,30,31]. While elevated androgens, such as testosterone, contribute to hyperandrogenic symptoms like hirsutism and acne in women with CS, they do not fully explain these clinical manifestations [35]. No male participants in the studies experienced adverse events related to increased androgens or estrogens. Among the female participants, hirsutism (12/106; 11%), acne (12/106; 11%), and hypertrichosis (1/106; 1%) were reported, all of mild to moderate severity (grade 1–2), and none led to study discontinuation [26]. Adverse events related to increased adrenal androgens and mineralocorticoid precursors may occur in 42.3–58.4% of patients receiving osilodrostat [14,21,27,57].

Compared to other drugs used in the treatment of CS, such as ketoconazole and levoketoconazole, no liver-related adverse events associated with osilodrostat have been reported in previous studies [26,27,31,49,58]. In the LINC 3 study, five (4%) enrolled patients experienced an increase in alanine aminotransferase (ALT) or aspartate aminotransferase (AST) levels above three times the upper LN. However, these elevations were typically mild and resolved spontaneously or after dose adjustment [26]. In contrast, another study, in which a relatively lower osilodrostat dosage was used, found no increase in transaminases during osilodrostat therapy [59].

QT prolongation on electrocardiogram, cardiac flutter, a type of supraventricular tachyarrhythmia and ventricular extrasystoles were among the 83 probable ADRs. Although cardiac flutter and ventricular extrasystoles were reported relatively infrequently, they displayed positive signals and were not included in the drug leaflet, highlighting their potential relevance as new ADR. In contrast, QT prolongation on electrocardiogram was a more commonly reported PT in FAERS and in clinical studies. QTc prolongation is an expected side effect of steroidogenesis inhibitors, necessitating close monitoring [51]. Clinical studies have demonstrated a dose-dependent relationship between osilodrostat and QT interval prolongation [12], which may contribute to cardiac arrhythmias. In the LINC 3, LINC 4, and LINC 6 studies, adverse events related to arrhythmogenic potential and QTc prolongation were observed in six patients (4.4%), three patients (4.1%), and two patients (2.1%), respectively [26,31,43]. In LINC 3, osilodrostat dose adjustments or temporary interruptions were required in three patients, with one patient discontinuing treatment [26]. Conversely, all cases in LINC 4 resolved without treatment discontinuation [31]. These adverse events remained uncommon during long-term therapy [27,30]. Furthermore, a retrospective analysis of eight patients with CS treated with osilodrostat reported a progressive increase in the mean QTc interval, reaching 455 ms (±23 ms SEM) after 12 weeks [59]. On the other hand, hypokalemia and other electrolyte imbalances may also contribute to lengthening of QT interval [60]. Additionally, both hypokalemia and hyperkalemia can manifest with various arrhythmic patterns, including premature ventricular complexes, ventricular fibrillation, ventricular tachycardia, atrial fibrillation and atrial flutter [61,62].

Furthermore, QT prolongation predisposes patients to premature ventricular contractions and is a recognized risk factor for ventricular extrasystoles, torsades de pointes [63] and atrial fibrillation [64,65]. Therefore, osilodrostat’s effects on electrolyte balance—including hyperkalemia or hypokalemia—and on QT prolongation may contribute to the development of ventricular extrasystoles and other ventricular arrhythmias, as well as the onset of cardiac flutter, particularly in susceptible individuals. Patients with CS frequently exhibit structural and functional cardiac changes [66,67] which could further predispose them to arrhythmias. Consequently, we may consider that cardiac flutter—most likely atrial flutter—may develop in patients with CS treated with osilodrostat, albeit less frequently, due to a complex interplay of electrolyte disturbances, autonomic imbalance [68], atrial and cardiac structural abnormalities, and QT prolongation.

Patients with CS and pre-existing cardiovascular disease or electrolyte imbalances who are treated with osilodrostat may be at higher risk of arrhythmias.

Cortisol plays a crucial role in modulating the immune response. CS, or chronic hypercortisolism, induces various immune system alterations [69], often leading to severe clinical complications such as sepsis and opportunistic infections, including fungal infections and COVID-19, which showed positive signals in our analysis. Prolonged exposure to high glucocorticoid levels, circadian rhythm disturbances, and CS associated comorbidities—such as obesity, hypertension, and diabetes, which are known risk factors for severe COVID-19 outcomes—all contribute to profound immune dysregulation in affected patients [69,70].

However, AI can also increase infection risk and worsen SARS-CoV-2 outcomes due to weakened innate immunity, characterized by increased monocytes and decreased cytotoxic natural killer cells [71,72]. A substantial decrease in cortisol levels caused by osilodrostat treatment can result in AI, potentially compromising the body’s ability to generate an adequate immune response. This weakened immune state may elevate the risk of opportunistic infections, such as fungal infections, sepsis, and COVID-19. In March 2020, the year osilodrostat received FDA and EMA approval for the treatment of CD and CS, respectively, the severe acute respiratory syndrome coronavirus 2 (SARS-CoV-2) pandemic emerged, leading to a high global mortality rate [73]. In clinical studies, infections such as urinary tract infections, influenza, and nasopharyngitis have been reported with frequencies ranging between 10 and 20% [3,13,74]. Nonetheless, no other specific infections have been consistently observed [12].

Positive signals were also observed for muscular weakness, rib fractures, and spinal fractures. However, these adverse events were not included in the drug leaflet as compared to the well-known reported arthralgia, back pain, and myalgia. Rib and spinal fractures in patients treated with osilodrostat are likely due to a combination of pre-existing osteoporosis and impaired bone quality, along with proximal muscle weakness and sarcopenia—particularly affecting the paraspinal and postural muscles—associated with CS [75,76]. Even after cortisol normalization with osilodrostat, bone fragility and muscle weakness may persist [77,78], leading to a predisposition to vertebral and rib fractures. The aforementioned factors may be further exacerbated or compounded by persistent hormonal imbalances-including suppression of gonadal and somatotroph axes in CS [75], as well as by rapid cortisol reduction during treatment with osilodrostat. The latter is relevant, as cortisol plays a complex, dose-dependent role in bone metabolism and an optimal cortisol level is essential for osteoblast differentiation [79], and bone remodeling [80]. Hence, we may speculate that marked cortisol suppression, whether due to AI [81] or excessive steroidogenesis inhibition, could impair osteoblast maturation and disrupt bone turnover, ultimately leading to reduced bone formation, compromised bone integrity, and an increased risk of fractures. Additionally, while clinical trials included only patients with CD, FAERS collects adverse events from patients with all types of CS, some of whom may have had longer disease duration and drug exposure. Notably, patients with EAS [82,83] and those with ACC undergoing mitotane [84] have a higher fracture risk than other individuals with endogenous CS. Furthermore, some patients may develop GWS during osilodrostat treatment, which includes symptoms such as fatigue, weakness, and joint pain. These effects could impair mobility and increase the risk of falls and fracture in frail individuals.

The higher probability of muscular weakness, rib fractures, and spinal fractures reported in FAERS suggests that these adverse events may emerge in real-world clinical practice. Our study underscores the importance of further investigation and ongoing monitoring of bone and muscle health, electrolytes, ECG, and hemodynamic parameters in patients receiving osilodrostat to enable early detection and management of these potential adverse events.

Depression, mood dysregulation, and sleep disturbances are observed in CS, including middle insomnia (patients awakening at least once during the night) in 69% of cases, late insomnia (patients awakening earlier than desired in the morning) in 57%, and early insomnia (inability to fall asleep at bedtime) in 29% [85,86,87,88]. On the other hand, prevalent symptoms of GWS also include sleep disturbances (29%) and mood changes (19%), alongside myalgias and arthralgias (50%), fatigue (45%), and weakness (34%). Thus, the rapid reduction in cortisol levels by osilodrostat can induce withdrawal-like symptoms, including mood swings and sleep disorders [89].

Finally, our analysis suggests that osilodrostat has a higher probability of being associated with adrenal gland cancer or lung neoplasm compared to other drugs. Several factors could explain this association. For instance, both adrenal gland cancer and EAS can be treated off-label with osilodrostat, and the latter (EAS) is often caused by small-cell lung cancer or bronho-pulmonary neuroendocrine tumors [35]. Moreover, some patients may have received osilodrostat for SH before the primary tumor was identified, with the cancer diagnosis emerging during treatment. It is important to note, however, that osilodrostat has only been available since 2020, which is a relatively short period for cancer development, and thus it is unlikely that these neoplasms are directly caused by the drug. Additionally, FAERS lacks detailed patient histories, meaning the reported cases may involve other cancer risk factors (e.g., smoking, genetic predisposition, associated diseases) that are not accounted for. While these findings indicate that these events are more frequently reported in patients receiving osilodrostat, the higher probability of reporting does not establish causality. These signals simply reflect a higher frequency of adverse event reports in comparison to other drugs.

### Limitations of Study

Early signal detection from post-marketing studies plays a crucial role in patient safety. This study is the first to investigate osilodrostat using reports of suspected ADRs from FAERS, queried via the OpenVigil 2.1 tool. However, our findings are subject to limitations inherent in pharmacovigilance studies. First, the data analyzed were collected from FAERS, a system based on spontaneous reporting of adverse reactions associated with real-world drug use. Since FAERS allows direct submissions, the quality of the information cannot be guaranteed, and inaccuracies may exist in the reported data since not all the cells are mandatory to be filled (e.g., age, sex). Even though many reports do not specify the sex of the patient, the prevalence of CD is consistent with our findings regarding male-to-female ratio. Reports come from diverse sources, including healthcare professionals (physicians, pharmacists, nurses), pharmaceutical companies, and the general public (patients, relatives), leading to variability in reporting accuracy. FAERS lacks quantitative information, such as denominator, making it difficult to determine the true incidence of adverse reactions. Underreporting is also a well-recognized limitation, further complicating incidence estimates. A reduced number of reports can trigger the small number effect that can induce potentially misleading signal strength, thus introducing interpretation bias. Finally, data mining alone cannot establish a direct causal relationship between a drug and an adverse reaction but highlights the need for continued safety monitoring in clinical practice.

## 5. Conclusions

The present analysis offers an overview of FAERS spontaneous reports on suspected ADRs in patients treated with osilodrostat, reinforcing its overall safety profile in clinical practice. Our study confirmed well-documented safety concerns, such as fatigue, nausea, AI, and headache, which have been reported in both pivotal clinical trials and observational studies. Additionally, we identified new potential safety signals involving ADRs not mentioned in the summary of product characteristics or package insert, warranting further investigation. These include cardiac flutter, ventricular extrasystoles, muscular weakness, rib and spinal fractures, sepsis, fungal infections, and COVID-19. However, as this is a preliminary analysis with inherent limitations, further in-depth research and validation are necessary to assess the clinical relevance and authenticity of these risks. Notably, the higher probability of these events compared to other drugs suggests a possible association with osilodrostat use. Nevertheless, these findings offer valuable insights and a foundation for future, more comprehensive safety assessments.

## Data Availability

The data are contained within the article.

## Supplementary Materials

The following supporting information can be downloaded at https://www.mdpi.com/article/10.3390/jcm14103518/s1: Table S1: 2 × 2 contingency tables. Table S2: Statistics indicators. Table S3: Frequency of adverse events by SOCs. Table S4: Adverse events that could be considered likely adverse reaction.
