# Supplementary material for: Osilodrostat Safety Profile: Findings from Real-World Data in the FAERS Database
Source: J Clin Med. 2025 May 17;14(10):3518. doi: 10.3390/jcm14103518 (PMC12112102; doi:10.3390/jcm14103518)
Supplement: Supplementary file 1 [file jcm-14-03518-s001.zip › jcm-3578347-supplementary.pdf]

Supplementary Material

Table S1. 2x2 contingency table.

	Drug of interest	All other drugs	Total
PT of interest	DE	dE	E
All other PTs	De	de	e
Total PTs	D	d	N

DE – cases where the PT of interest occurred when the drug of interest was used;
dE – cases where the PT of interest occurred when other drugs were used;
E – total cases of the PT of interest (E = DE + dE);
De – cases where all other PTs occurred when the drug of interest was used;
de – cases where all other PTs occurred when all other drugs were used;
e – total cases of other PTs (e = De + de);
D – total cases reported for the drug of interest (D = DE + De);
d – total cases reported for all other drugs (d = dE + de);
N – total cases in the dataset (N = D + d or N = E + e).

Table S2. Statistics indicators

Statistic indicator	Formula
Reporting rate (R)	$R = \frac{DE}{D}$
Proportional Reporting Ratio (PRR)	PRR = (DE/E)/(De/e)
Chi-square (χ^2)	χ^2= [(DE×de− De×dE)^2(DE+dE+De+de)]/[D×d×E×e]
Reporting odds ratio (ROR)	$ROR = \frac{DE \times de}{De \times dE}$
95% confidence interval (95% CI)	exp (ln (ROR) − 1.96 × SE{ln(ROR)}) to<br>exp (ln(ROR) + 1.96 × SE{ln(ROR)})

Table S3. Frequency of PTs by SOCs

SOC	PTs occurrences	
	n	%
General disorders and administration site conditions	457	18.4%
Injury, poisoning and procedural complications	311	12.6%
Gastrointestinal disorders	278	11.2%
Investigations	260	10.5%
Nervous system disorders	184	7.4%
Musculoskeletal and connective tissue disorders	138	5.6%
Endocrine disorders	116	4.7%
Infections and infestations	105	4.2%
Metabolism and nutrition disorders	87	3.5%
Psychiatric disorders	83	3.3%
Skin and subcutaneous tissue disorders	80	3.2%
Respiratory, thoracic and mediastinal disorders	65	2.6%

Vascular disorders	62	2.5%
Surgical and medical procedures	58	2.3%
Neoplasms benign, malignant and unspecified (incl cysts and polyps)	54	2.2%
Cardiac disorders	46	1.9%
Renal and urinary disorders	39	1.6%
Eye disorders	12	0.5%
Hepatobiliary disorders	12	0.5%
Reproductive system and breast disorders	11	0.4%
Immune system disorders	9	0.4%
Pregnancy, puerperium and perinatal conditions	7	0.3%
Blood and lymphatic system disorders	3	0.1%
Ear and labyrinth disorders	3	0.1%
Social circumstances	1	0.0%
**Total**	2481	100.0%

Table S4. Preferred terms that could be considered likely adverse reaction. DE – Cases where the adverse event of interest occurred when the drug of interest was used; PRR – proportional reporting rate; PT – preferred term; R – reporting rate; SOC – system organ classes

SOC	Number of PTs	Total cases	PT	DE	R	PRR	Chi_ squared
Cardiac disorders	2	8	cardiac flutter	4	0.5%	18.42	49.57
			ventricular extrasystoles	4	0.5%	11.85	29.62
Endocrine disorders	6	80	adrenal insufficiency	53	6.8%	147.92	7523.98
			adrenocortical insufficiency acute	9	1.2%	154.92	1215.39
			glucocorticoid deficiency	3	0.4%	248.13	504.37
			hyperadrenocorticism	8	1.0%	621.28	4195.85
			mineralocorticoid deficiency	3	0.4%	5513.88	8611.49
			steroid withdrawal syndrome	4	0.5%	112.91	336.54
Eye disorders	1	3	glaucoma	3	0.4%	4.70	5.43
Gastrointestinal disorders	5	153	abdominal discomfort	14	1.8%	2.45	10.72
			abdominal distension	13	1.7%	4.10	27.58
			appendicitis	3	0.4%	10.39	16.93
			nausea	90	11.5%	3.44	158.32
			vomiting	33	4.2%	2.18	20.34
General disorders and administration site conditions	12	247	asthenia	37	4.7%	3.16	53.25
			decreased appetite	37	4.7%	4.98	114.86
			disease progression	14	1.8%	3.14	18.43
			fatigue	99	12.7%	3.92	219.17
			hunger	3	0.4%	7.53	11.08
			night sweats	5	0.6%	5.31	13.47
			oedema	9	1.2%	5.27	27.05
			oedema peripheral	11	1.4%	2.75	10.58
			peripheral swelling	17	2.2%	3.84	33.05
			swelling face	6	0.8%	2.91	5.73
			therapeutic product effect prolonged	5	0.6%	139.01	549.41
			thirst	4	0.5%	6.83	14.50

Infections and infestations	4	57	cOVID-19	32	4.1%	5.07	101.16
			fungal infection	4	0.5%	3.67	5.33
			sepsis	9	1.2%	2.63	7.56
			urinary tract infection	12	1.5%	2.37	8.25
Injury, poisoning and procedural complications	4	221	contusion	7	0.9%	2.37	4.25
			off label use	182	23.3%	6.23	825.10
			product administration interrupted	8	1.0%	30.68	200.69
			product use in unapproved indication	24	3.1%	2.86	27.49
Investigations	18	178	blood corticotrophin increased	10	1.3%	1140.80	9613.79
			blood glucose increased	16	2.0%	2.38	11.57
			blood magnesium decreased	4	0.5%	16.33	43.21
			blood potassium abnormal	3	0.4%	37.71	73.48
			blood potassium decreased	22	2.8%	25.06	484.23
			blood potassium increased	9	1.2%	17.73	125.77
			blood pressure decreased	8	1.0%	3.82	13.97
			blood pressure increased	15	1.9%	3.09	19.24
			blood sodium decreased	8	1.0%	13.88	83.14
			blood testosterone increased	4	0.5%	57.24	167.79
			cortisol abnormal	4	0.5%	529.33	1564.03
			cortisol decreased	31	4.0%	594.20	17154.68
			cortisol free urine increased	4	0.5%	2005.05	5470.30
			cortisol increased	12	1.5%	497.49	5300.54
			heart rate increased	10	1.3%	3.13	12.51
			heart rate irregular	4	0.5%	5.36	10.17
			electrocardiogram qt prolonged	7	0.9%	2.88	6.81
			hepatic enzyme increased	7	0.9%	3.29	9.00
Metabolism and nutrition disorders	7	64	blood glucose decreased	7	0.9%	4.16	13.84
			dehydration	14	1.8%	3.62	24.07
			fluid retention	7	0.9%	4.31	14.68
			hyperkalaemia	6	0.8%	5.63	18.48
			hypokalaemia	10	1.3%	7.52	50.29
			hyponatraemia	5	0.6%	2.92	4.53
			weight increased	15	1.9%	2.07	7.29
Musculoskeletal and connective tissue disorders	6	77	arthralgia	29	3.7%	2.25	19.21
			back pain	17	2.2%	2.34	11.89
			muscular weakness	8	1.0%	2.25	4.38
			myalgia	16	2.0%	2.78	16.59
			rib fracture	4	0.5%	6.66	13.99
			spinal fracture	3	0.4%	4.66	5.35
Neoplasms benign, malignant and unspecified (incl cysts and polyps)	4	27	adrenal gland cancer	5	0.6%	274.78	1085.84
			lung neoplasm	3	0.4%	18.33	33.31
			neoplasm malignant	12	1.5%	4.01	24.28
			neoplasm progression	7	0.9%	4.77	17.27
	3	66	head discomfort	3	0.4%	5.62	7.23

Nervous system disorders			headache	51	6.5%	2.44	43.07
			loss of consciousness	12	1.5%	2.82	12.40
Psychiatric disorders	2	11	mood swings	4	0.5%	3.39	4.57
			sleep disorder	7	0.9%	3.06	7.79
Renal and urinary disorders	1	9	nephrolithiasis	9	1.2%	6.29	34.97
Skin and subcutaneous tissue disorders	4	15	acne	4	0.5%	3.34	4.43
			hair growth abnormal	4	0.5%	15.96	42.10
			hirsutism	4	0.5%	86.15	255.57
			hypertrichosis	3	0.4%	50.74	100.47
Surgical and medical procedures	2	23	hospitalisation	20	2.6%	3.36	31.14
			hysterectomy	3	0.4%	10.94	18.06
Vascular disorders	2	33	hypertension	15	1.9%	2.22	8.96
			hypotension	18	2.3%	2.82	19.46
